# The Effect of Nutrition Therapy and Exercise on Cancer-Related Fatigue and Quality of Life in Men with Prostate Cancer: A Systematic Review

**DOI:** 10.3390/nu9091003

**Published:** 2017-09-12

**Authors:** Brenton J. Baguley, Kate A. Bolam, Olivia R. L. Wright, Tina L. Skinner

**Affiliations:** 1 School of Human Movement and Nutrition Sciences, The University of Queensland, Brisbane, QLD 4072, Australia; Kate.Bolam@ki.se (K.A.B.); o.wright@uq.edu.au (O.R.L.W.); t.skinner@uq.edu.au (T.L.S.); 2 Department of Neurobiology, Care Sciences and Society, Division of Nursing, Karolinska Institute, 171 77 Stockholm, Sweden; 3 Mater Medical Research Institute, The University of Queensland, Brisbane QLD 4072, Australia

**Keywords:** nutrition, exercise, prostate cancer, cancer-related fatigue, quality of life

## Abstract

Background: Improvements in diet and/or exercise are often advocated during prostate cancer treatment, yet the efficacy of, and optimal nutrition and exercise prescription for managing cancer-related fatigue and quality of life remains elusive. The aim of this study is to systematically review the effects of nutrition and/or exercise on cancer-related fatigue and/or quality of life. Methods: A literature search was conducted in six electronic databases. The Delphi quality assessment list was used to evaluate the methodological quality of the literature. The study characteristics and results were summarized in accordance with the review’s Population, Intervention, Control, Outcome (PICO) criteria. Results: A total of 20 articles (one diet only, two combined diet and exercise, and seventeen exercise only studies) were included in the review. Soy supplementation improved quality of life, but resulted in several adverse effects. Prescribing healthy eating guidelines with combined resistance training and aerobic exercise improved cancer-related fatigue, yet its effect on quality of life was inconclusive. Combined resistance training with aerobic exercise showed improvements in cancer-related fatigue and quality of life. In isolation, resistance training appears to be more effective in improving cancer-related fatigue and quality of life than aerobic exercise. Studies that utilised an exercise professional to supervise the exercise sessions were more likely to report improvements in both cancer-related fatigue and quality of life than those prescribing unsupervised or partially supervised sessions. Neither exercise frequency nor duration appeared to influence cancer-related fatigue or quality of life, with further research required to explore the potential dose-response effect of exercise intensity. Conclusion: Supervised moderate-hard resistance training with or without moderate-vigorous aerobic exercise appears to improve cancer-related fatigue and quality of life. Targeted physiological pathways suggest dietary intervention may alleviate cancer-related fatigue and improve quality of life, however the efficacy of nutrition management with or without exercise prescription requires further exploration.

## 1. Introduction

Advances in treatment for prostate cancer have improved prostate cancer-specific survival, with 5–10-year disease-free survival rates in Western countries now between 75% and 94% [1,2,3,4,5]. Androgen deprivation therapy (ADT), radiotherapy, chemotherapy, and surgery (radical prostatectomy) are the current mainstream treatment options, due to their efficacy in reducing prostate-specific disease progression [6]. However, side effects from prostate cancer treatment are highly prominent; hence recent clinical focus has turned to managing or reducing treatment-related side effects [7,8,9,10]. Cancer-related fatigue (CRF) is the most commonly reported treatment-related side effect from prostate cancer [11,12,13], and may impact quality of life during, and for many years following treatment [14,15]. Targeted diet and exercise prescription for men with prostate cancer is thought to offer a long-term, multi-health benefit for managing CRF [16,17]. However, whether diet and/or exercise interventions are indeed effective in improving CRF requires systematic exploration. Further, to enable effective translation to clinical practice, the optimal nutrition management and exercise prescription principles of frequency, intensity, time and type for improving CRF need to be elucidated.

Diet and nutritional status are thought to be pivotal for reducing the risk of prostate cancer-specific mortality [18], and managing other clinical endpoints such as CRF and quality of life [10,19]. Evidence-based nutritional guidelines for prostate cancer advocate for the maintenance of body mass, lean body composition and individual nutrient requirements for reducing the onset of other prevalent chronic diseases [20,21,22]. However, evidence-based nutritional guidelines for alleviating treatment-specific side effects, such as CRF and quality of life, are limited. Over the continuum of prostate cancer treatment, particularly with the use of ADT, a profound degenerative impact is seen on body composition (reduced lean mass, increased fat mass and total body mass) and systemic inflammation (increased interleukin (IL)-6, IL-8, C-reactive protein (CRP), and tumour necrosis factor-α (TNF-α)) [23,24]. These effects are thought to, at least in part, explain the aetiology of CRF, providing a targeted approach for dietary intervention to reduce CRF and improve quality of life in men with prostate cancer. With approximately 70% of prostate cancer survivors currently overweight or obese [25], and 15.1% of survivors not meeting the nutrition guidelines for fruits and vegetables [26], the lifestyle choices of men in westernised countries may predispose them to CRF. Specific modification to dietary intake may offer attenuations to the body mass, composition and inflammatory responses to treatment [27,28,29]; however, systematic exploration of the efficacy of dietary management for CRF and improving quality of life remains unknown. Thus, it is imperative to understand the role of diet patterns and isolated nutrients for managing CRF and quality of life to offer an evidence-based approach for the nutrition management of CRF.

The exercise oncology guidelines suggest a combination of both aerobic and resistance training performed 3–5 times per week, at a moderate intensity, for 20–30 min for all cancer patients [30]. Cross-sectional analysis suggests 12–50% of the prostate cancer population currently meet the exercise oncology guidelines [31,32]; indicating the majority of men with prostate cancer are not benefiting from the health-benefits associated with exercise. Both aerobic and resistance training modes of exercise have been shown to improve CRF and quality of life in men with prostate cancer [33,34,35,36,37,38]; however, beyond the mode of exercise, it is unknown if exercise frequency, intensity and duration are pivotal to the benefits seen from exercise on CRF and quality of life in prostate cancer. Moderate-intensity exercises improve CRF in cancer survivors [39], yet investigations on the effects of different exercise intensities in prostate cancer are yet to be systematically explored. Thus, the optimal exercise prescription to reduce CRF and improve quality of life is yet to be elucidated. Understanding the characteristics of optimal exercise management for men with prostate cancer may provide a more informed and impactful clinical care management of CRF and quality of life.

Nutrition therapy with exercise prescription may offer a combined targeted approach to alter the key pathways underlying CRF [40], and subsequently improve quality of life. Whilst isolated improvements in diet and exercise are thought to be advantageous during and after prostate cancer treatment [41], the combined effects of nutrition and exercise on CRF and quality of life in men with prostate cancer is yet to be reviewed. The aim of this study was to systematically review the literature from controlled and single arm intervention trials that investigated the effects of nutrition, exercise or combined nutrition and exercise on CRF and/or quality of life, in men diagnosed with prostate cancer. It is anticipated the findings from this review will inform future research, as well as dieticians, nutritionists, other allied health professionals and medical specialists on the adjunctive effects of diet and/or exercise on CRF and quality of life management in men with prostate cancer.

## 2. Methods

### 2.1. Literature Search

The systematic review was conducted in accordance with the Preferred Reporting Items for Systematic reviews and Meta Analyses (PRISMA) statement [42]. Electronic databases searched in September 2016 included PubMed, PEDro, EMBASE (via EMBASE.com), Proquest, CINAHL, and CENTRAL (Cochrane Central Register of Controlled Trials). Search terms included combinations of thesaurus terms (MeSH in PubMed and CENTRAL, EMtree in EMBASE) and free text terms. Free text terms for prostate cancer (prostatic neoplasms, prostat* neoplasms, prostate cancer, prostat* cancer, prostat* tumor *, prostat* tumour*, prostat* carcino*) were used in AND-combination with search terms specific to exercise (exercis*, training, weight*, aerobic, strength*, physical activit*, endurance, walk* run*, jog*) AND-combination search terms representing a nutrition intervention (diet*, nutrition*, food*, nutr*, supplement*) and search terms representing cancer-related fatigue and quality of life (fatigue, tired*, quality of life, inflamm*, anti-inflamm*, wellbeing, well-being, function, physical capacity). In PubMed, search results were limited by search terms indicating specific study design (e.g., trial, random*, intervention, study, control*, program, pilot study). The complete list of search terms is available on request. 

### 2.2. Inclusion Criteria

The inclusion criteria were specified by the Population, Intervention, Control, Outcomes, Study design (PICOS) framework as seen in Table 1. This included (1) population: men with a histologically confirmed diagnosis of prostate cancer (including all stages of, and treatments for, prostate cancer), and studies of varied cancer populations if results from men with prostate cancer were reported separately; (2) intervention: any structured diet only (any dietary modification), exercise only (any structured prescription of exercise detailing frequency, intensity, time and type of exercise), or any combined diet and exercise protocol, for any duration (with the exception of a single bout of exercise comparing pre- and post-exercise acute fatigue measures); (3) control: comparison group receiving diet, exercise, or a combined diet and exercise of a lesser intensity, control group not receiving the intervention at any time point during the trial, waiting list control care, or crossover data of double cross over randomized controlled trials only taken at midpoint (4) outcomes: cancer-related fatigue measured by Functional Assessment of Cancer Therapy (FACT)-Fatigue, FACT-General, FACT-Prostate, Multidimensional Fatigue Inventory-Short Form (MFI-SF), Schwartz Cancer Fatigue Scale (SCFS), Piper Fatigue Scale (PFS), Brief Fatigue Inventory (BFI), quality of life as measured by the Medical Outcomes Study: 36-Item Short Form Survey (MOS SF-36), European Organisation of Research and Treatment of Cancer Quality of Life Questionnaire (EORTC QLQ-C30), EORTC Prostate-specific module (PR25), or any other measure of cancer-related fatigue or quality of life study design; and (5) study design: randomised controlled trials (RCT), controlled trials (CT) or single group cohort studies. Only full-text English articles of human trials, published in peer-reviewed journals were included in the search process. 

Titles and abstracts of articles identified through the search process were first reviewed to exclude articles out of scope. B.J.B., T.L.S. and K.A.B. independently screened the full texts to identify eligible articles. Disagreements were discussed and resolved. Articles that met the inclusion criteria were examined to ensure that data from the same participants within the same study were not reported in more than one article. The data extraction procedure followed the PRISMA statement [42]. Reference lists of eligible articles were manually checked for additional references.

### 2.3. Data Extraction and Quality Assessment

Details of (1) participant and study characteristics; (2) the nutrition and/or exercise intervention; (3) and study results were independently extracted by two authors (B.J.B. and K.A.B.). Nutrition interventions were defined as any structured dietary manipulations, with the exception of herbal, vitamin, mineral or any spurious nutrition supplementation to isolate the efficacy of whole food dietary changes on CRF and quality of life. Exercise interventions were defined as any exercise programmes with a stipulated session frequency (number of exercise sessions per week), intensity e.g., percentage of peak heart rate or age predicted maximum heart rate (APMHR) [43], time (exercise session duration), and type (eligible articles could include aerobic exercise, resistance training or a combination of these types of exercise). Any intervention that also incorporated strategies that may have influenced quality of life, e.g., meditation, relaxation, stress, or mood management in combination with a diet and/or exercise intervention, where the effects of the nutrition therapy and/or exercise prescription could not be isolated, were excluded. Methodological quality of the included articles was independently reviewed by two authors (B.J.B. and K.A.B.), using the modified Delphi assessment list [44]. Three assessment criteria were excluded from the original Delphi assessment list (blinding of the trainers, blinding of the outcome assessors and blinding of the participants), as the outcome measures required responses that could not be influenced by interpretations of the assessor, and like most diet and/or exercise interventions, it is difficult to blind participants and trainers to the fact they are following a nutrition plan and/or exercising. 

The modified Delphi quality assessment list included the following six items: (1) was a method of randomisation performed; (2) was the treatment allocation concealed prior to baseline testing; (3) were the groups similar at baseline for the measures of fatigue and quality of life, and if groups were not similar at baseline, this was adjusted for in subsequent analysis; (4) were the eligibility criteria specified; (5) were point measures and measures of variability presented for measures of fatigue or quality of life; and (6) did the analysis include an intention-to-treat analysis (defined as all participants randomised included in analysis). For variability, this was often operationally defined as ‘data describing the central tendency and variability at each measured time point were reported in the study’, and intention-to-treat analysis operationally defined, as ‘all participants randomised at baseline were included in subsequent endpoint analysis’. The criteria were rated equally using a ‘yes’ (1), ‘no’ (0), or ‘unclear’ (0) answer format. A quality score was generated as a percentage of the maximum score for each included study. 

## 3. Results

### 3.1. Study Design and Research Quality

**Figure 1:**
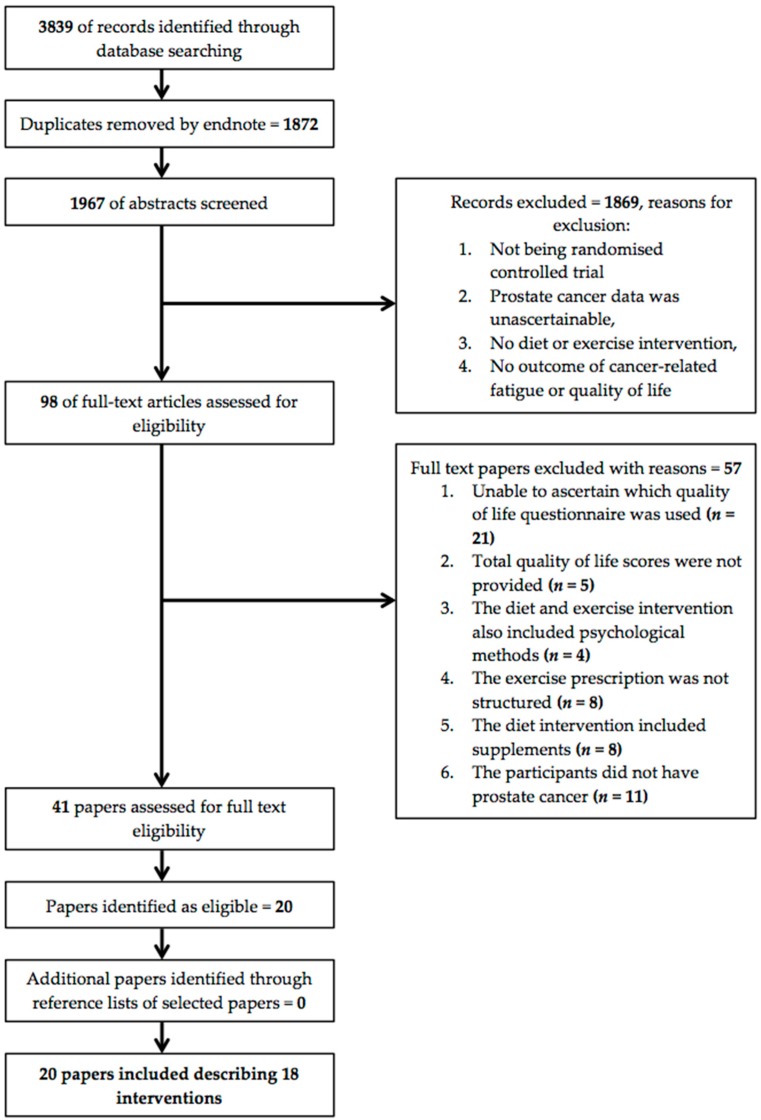
Search process flow chart.

The systematic search resulted in 3839 records; details of the search process are shown in Figure 1. Abstracts of these records were assessed for eligibility; full texts of the remaining 41 articles were independently examined by B.J.B., K.A.B., and T.L.S. A total of 20 studies met the inclusion criteria; including 19 randomised controlled trials [16,17,45,46,47,48,49,50,51,52,53,54,55,56,57,58,59,60,61], and one prospective controlled trial [62] informed this systematic review. One study included a diet only intervention [56], three studies included combined diet and exercise interventions [16,17,52], and sixteen included exercise only interventions [45,46,47,48,49,50,51,53,54,55,57,58,59,62]. Two exercise only interventions were described as two separate studies, both by Galvao et al. [47,48,60,61]. Different quality of life measures were described in each study and thus warranted inclusion, however for the purpose of this systematic review the four studies were treated as two interventions. 

### 3.2. Quality Assessment

Results of the methodological quality assessment are presented in Table 2. Methodological quality scores ranged from 33% [62] to 100% [16,45,46,47,48,51,53,54,55], with a mean of 80%. All of the studies specified eligibility criteria and all but four studies [17,56,57,62] provided point estimates and measures of variability. Despite the generally high methodological quality of the included interventions, an intention to treat approach was either not conducted or adjusted in seven of the 18 interventions [17,49,50,52,58,59,62]. 

### 3.3. Study Populations

A total of 1397 participants were included across the 18 interventions. Sample sizes ranged from 20 [46] to 155 [54] participants. The average age of participants ranged from 64.7 [49] to 73.1 years [46]. Eight studies included men receiving ADT only [16,17,45,48,52,53,54,56], seven studies included men receiving both ADT and radiation therapy [46,47,51,55,57,58,62], two studies included men receiving radiation therapy only [50,59], and one intervention included men who had either undergone surgery (radical prostatectomy), radiation therapy, or ADT [49]. Eleven studies reported the average duration that the participants had been receiving ADT at the time of enrolment, which ranged from 5.6 days [45] to 39.0 months [57]. 

### 3.4. Measures of Cancer-Related Fatigue

Fifteen interventions reported the effects of diet and/or exercise interventions on CRF [16,17,45,46,48,50,51,52,53,54,55,57,58,59,62]. No diet only interventions reported CRF outcomes. Three interventions reported the effects of combined diet and exercise on CRF, with two measuring CRF by FACT-F [16,17], and one using the MFI-SF [52]. Twelve exercise only interventions assessed CRF using the FACT-F (*n* = 5) [45,53,54,55,58], EORTC-C30 fatigue domain (*n* = 2) [48,51], BFI (*n* = 2) [59,62], MFSI-SF (*n* = 1) [46], SCFS (*n* = 1) [57], and PFS (*n* = 1) [50]. 

### 3.5. Measures of Quality of Life

Fourteen interventions reported the effects of diet and/or exercise interventions on quality of life [16,17,37,44,45,46,48,51,54,58,59,60,62,63]. The diet only RCT measured quality of life through both FACT-P and FACT-G questionnaires [56]. The two combined diet and exercise RCTs used the FACT-G [16] and FACT-P [16,17]. The eleven exercise only studies reported quality of life using either questionnaire of FACT-P (*n* = 4) [50,53,54,55], SF-36 (*n* = 4) [45,46,47,48], EORTC-C30 (*n* = 4) [47,49,51,58,60], FACT-G (*n* = 1) [55], and PORPUS (*n* = 1) [53]. 

### 3.6. Intervention Characteristics

#### 3.6.1. Diet only Interventions

The one diet only intervention investigated the effects of 12 weeks of supplementation with 20 g/day soy isoflavones, comprising 160 mg isoflavones [56]. 

#### 3.6.2. Combined Diet and Exercise Interventions

Characteristics of the combined diet and exercise interventions are shown in Table 3. The duration of these interventions ranged from 12 [16,17] to 24 weeks [52]. A Nutritionist or Dietician provided the dietary advice in all three interventions. One intervention consisted of a one-hour diet consultation [52], whilst the two other interventions consisted of fortnightly 20-min group-based nutrition seminars [16,17]. All three interventions provided participants with written nutrition material handouts, with one intervention individualising the nutrition information to address participants’ dietary preferences [52]. The nutrition therapy of two interventions emphasised reducing saturated fat, refined carbohydrates and increasing dietary fibre intake [16,17]. One intervention provided an individualised nutrition consult to align with the United Kingdom’s Dietary Guidelines [52]. 

Combined aerobic and resistance training was prescribed in two of the diet and exercise interventions [16,17], with one intervention prescribing aerobic exercise only [52]. Aerobic exercise sessions (via cycling, brisk walking, light jogging, or swimming) were performed once per week in two interventions [16,17], and three times per week in one intervention [52]. Aerobic exercise session duration was 30 min for all three interventions [16,17,52]. Whilst one intervention did not describe the intensity of exercise [52], moderate-vigorous intensity the other two interventions reported exercise intensity as 55–80% APMHR, with a rating of perceived exertion ranging from 11 to 13 (on a scale from 6 to 20) [16,17]. Moderate-hard intensity resistance training was progressively increased over the training load in two interventions [16,17], from two to four sets of 8–12 repetitions at 60% of one repetition maximum, with an intended rating of perceived exertion ranging from 7 to 12. Two of these interventions were partially supervised [16,17], whilst one intervention involved unsupervised home-based exercise [52].

#### 3.6.3. Exercise Only Interventions

Of the fourteen exercise only interventions, five interventions included a combination of aerobic and resistance training [45,47,48,49,58], two studies prescribed aerobic exercise only [50,62], five prescribed resistance training only [46,51,54,57,59] and two compared the effects of aerobic exercise versus resistance training in the same study [53,55]. Durations of the exercise interventions ranged from 8 [50] to 52-weeks [57]. Session frequency of the fourteen exercise interventions ranged from two [45,46,48] to five days per week [53,58]. Length of exercise session was reported in eleven interventions, and ranged from 20 to 60 min per session [45,46,47,48,49,50,53,55,58,59,62]. 

Aerobic exercise (in aerobic-only or combined aerobic with resistance training interventions) was prescribed at a moderate-vigorous intensity of either 60–85% of heart rate reserve [50,53], 50–85% maximal heart rate [45,47,48,55,58], and 40–70% APMHR [43]. All four aerobic-only exercise interventions utilised heart rate monitors during the intervention, yet only one study reported compliance to the prescribed intensity. Monga et al. [50] reported 88% of participants achieved a moderate-vigorous prescribed intensity. No intervention investigated the effect of low or high intensity aerobic exercise on CRF. 

Resistance training included free weights, assisted machine weights, and resistance bands, prescribed to target major muscle groups [45,47,48,49,58]. The exercise prescription ranged from four to eleven exercises, of which one to four sets of six to fifteen repetitions was prescribed [45,46,47,48,49,51,53,54,55,57,58,59]. Six interventions indicated the weight load prescription was between 60% [45,46,53,54,55,58] to 90% [51] of one repetition maximum, one intervention used resistance prescription as a percentage of body weight [57], whilst four interventions did not indicate the intensity of the resistance exercise (by percentage of BW or 1RM) [47,48,49,59]. Rating of perceived exhaustion was measured in one of six resistance training interventions, with Cormie, et al. [46] reporting moderate-vigorous intensity resistance training was performed at a rating of perceived exertion of 12–16.

Nine of the fourteen exercise only interventions reported sessions were supervised by qualified health professionals [45,46,47,48,49,50,51,55,59]. Two interventions were partially supervised [57,58], whilst one study offered group training [53], which was optional and the only supervision provided throughout the intervention. Habitual dietary intake was monitored in three exercise only interventions [45,46,58], yet dietary analysis was not reported in any intervention. 

### 3.7. Dropout, Attendance, and Adverse Events

The diet only intervention reported a 28% dropout rate [56]. All three diet and exercise interventions reported dropouts, ranging from 3% [52] to 15% [17]. All but one [58] of the fourteen exercise only interventions report dropouts; these ranged from 3 [47] to 42% [50]. 

Adherence to the diet only soy supplementation intervention was 72% [56]. All three combined diet and exercise interventions reported participant attendance. Attendance at the exercise component of the diet and exercise interventions ranged from 85% [17] to 95% [16], whilst attendance to the nutrition component of the intervention was not reported. One of the three combined diet and exercise interventions reported adherence to the exercise sessions using self-reported logbooks, with 64% of participants returning their logbooks [52]; however, informal fortnightly phone reviews indicated 91.5% of participants adhered to the exercise prescription. Exercise attendance was reported in twelve of the fourteen exercise only interventions [45,46,47,48,49,51,53,54,55,57,59,62], with participants attending 63% [59] to 96% [45] of all exercise sessions. 

The soy supplement diet only intervention reported 19 adverse events from the supplement [56], yet the nature of the adverse events was not reported. Adverse events were reported in two of the three combined diet and exercise interventions [16,17]. One participant was excluded from the exercise intervention due to previously undiagnosed cardiac problems [16], and one participant discontinued the exercise intervention after developing atrial fibrillation [17], however it is unknown if the atrial fibrillation was related to the exercise intervention. Ten of the fourteen exercise only interventions reported on adverse events [45,46,47,48,49,51,54,55,57,62], with three of the interventions reporting adverse events from the exercise intervention [47,51,55]. Musculoskeletal injuries were the most frequently reported adverse event to the exercise intervention [47,51,55], with knee and back injuries reported by five participants, and all cases ceasing participation in the respected exercise intervention. Two studies reported cardiac related adverse events from the exercise intervention [47,55]. Two participants from separate interventions suffered acute myocardial infarctions during exercise; both withdrew from the exercise intervention [47,55]. One participant withdrew from the exercise intervention because of chest pain, with subsequent cardiologic investigations revealing normal functioning [55]. 

### 3.8. Reported Findings

#### 3.8.1. Cancer-Related Fatigue

**Figure 2:**
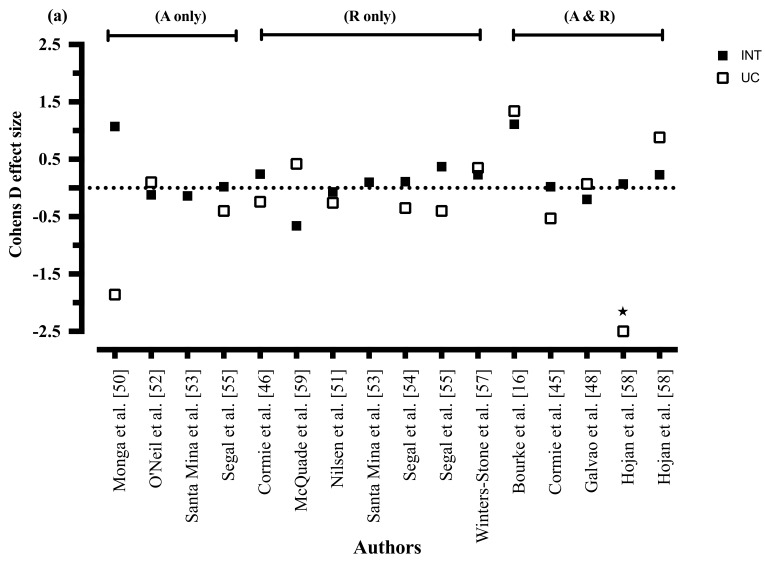

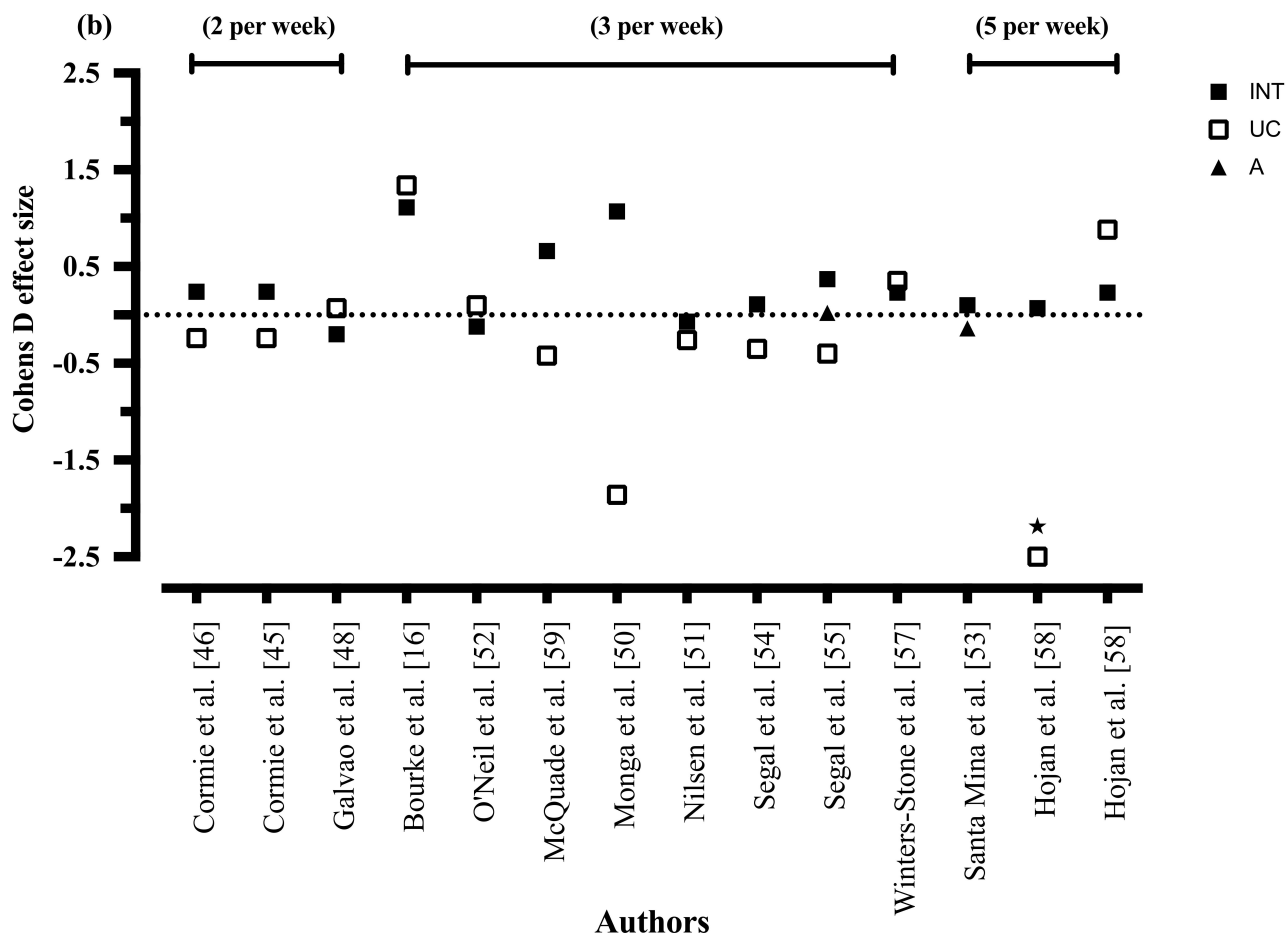

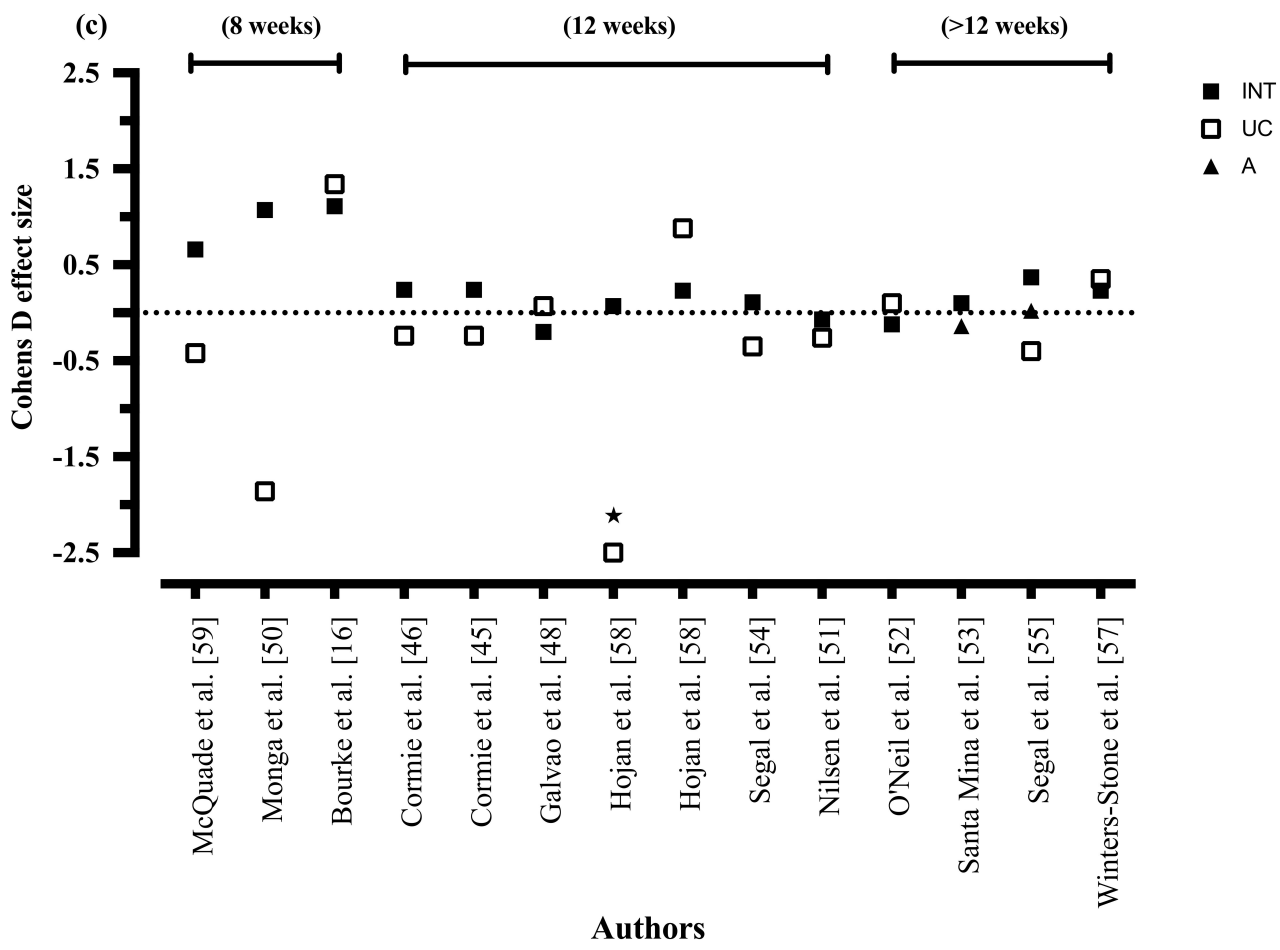
(**a**) Cohens D effect size of exercise mode on cancer-related fatigue; (**b**) Cohens D effect size of exercise frequency on cancer-related fatigue; (**c**) Cohens D effect size of exercise duration on cancer-related fatigue (A = aerobic exercise, R = resistance training, INT = intervention, UC = usual care, b = second measure of cancer-related fatigue, * = effect size of −6.92).

The effects of the interventions on CRF are shown in Table 4. Two of the three combined diet and exercise interventions showed between-group improvements in CRF compared to usual care [16,17], whilst five of twelve exercise only RCTs showed between-group improvements in CRF with exercise when compared to usual care [45,48,55,57,58]. Four of eleven exercise only RCTs showed significant within-group improvements in CRF from the exercise intervention [50,54,55,58]. The effect sizes of exercise mode, frequency, and duration from the intervention, compared to usual care, on CRF are shown in Figure 2. 

#### 3.8.2. Mode of Exercise

All three combined resistance and aerobic exercise RCTs showed between-group improvements in CRF when compared to usual care [45,48,58]. Two of six RCTs utilising resistance training alone showed between-group improvements in CRF when compared to usual care [55,57]. Zero of four aerobic interventions showed significant between-group improvements in CRF, with only one intervention reporting within-group improvements with exercise [50]. In addition, one aerobic exercise intervention reported between-group improvements in CRF at midpoint testing (12-weeks, *p* = 0.004) which were no longer significant at 24-weeks (*p* = 0.080) [55]. 

#### 3.8.3. Exercise Intensity

Five of nine moderate-vigorous (aerobic exercise) or moderate-hard (resistance training) intensity interventions showed between-group improvements in CRF when compared to a control group [45,48,57,58], or when resistance training was compared to aerobic exercise [55]. One study investigated light intensity training and found no between- or within-group improvements in CRF relative to usual care [59]. Similarly, an exercise study that utilised a varied exercise intensity (40–90% 1RM) showed no between- or within-groups improvements in CRF compared to usual care [51]. Neither light, high or sprint aerobic exercise, nor light, very hard or maximal resistance training exercise was trialled in any intervention investigating outcomes of CRF. 

#### 3.8.4. Exercise Duration

Zero of the two 8-week interventions [57,59] showed any between- or within-group improvements in CRF. Four of the eight studies that prescribed exercise for 12-weeks or longer showed between-group improvements in CRF compared to usual care [45,48,57,58], whilst one of three RCT showed between-group improvements in CRF at midpoint testing (12-week) [55]. One of two 24-week interventions showed between-group improvements, with resistance training demonstrating greater improvements than aerobic exercise [55]. One of two 52-week interventions found between-group improvements with exercise compared to usual care [57]. 

#### 3.8.5. Exercise Frequency

Two of three RCTs prescribing exercise twice a week showed between-group improvements in CRF compared to usual care [45,48]. Only two of eight studies that prescribed exercise three times per week showed between-group improvements in CRF compared to usual care [55,57]. The one study exploring exercise performed five times per week showed between-group improvements in CRF compared to usual care [58]. 

#### 3.8.6. Quality of Life

**Figure 3:**
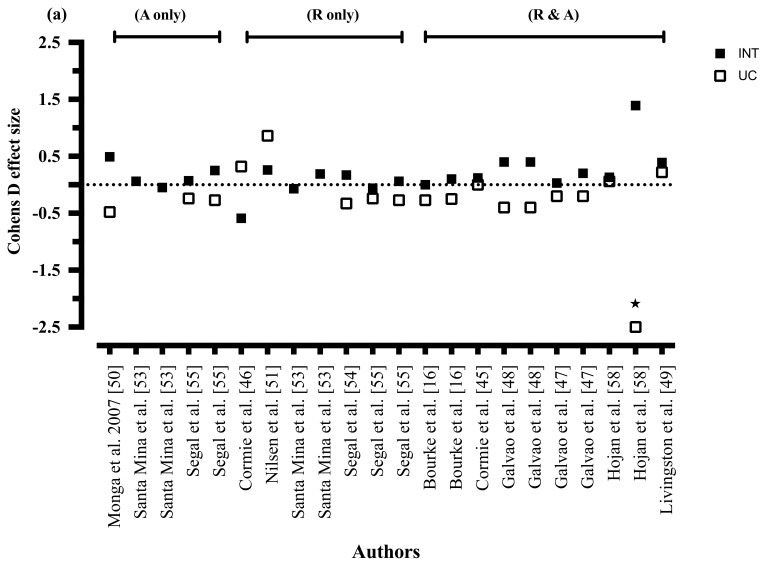

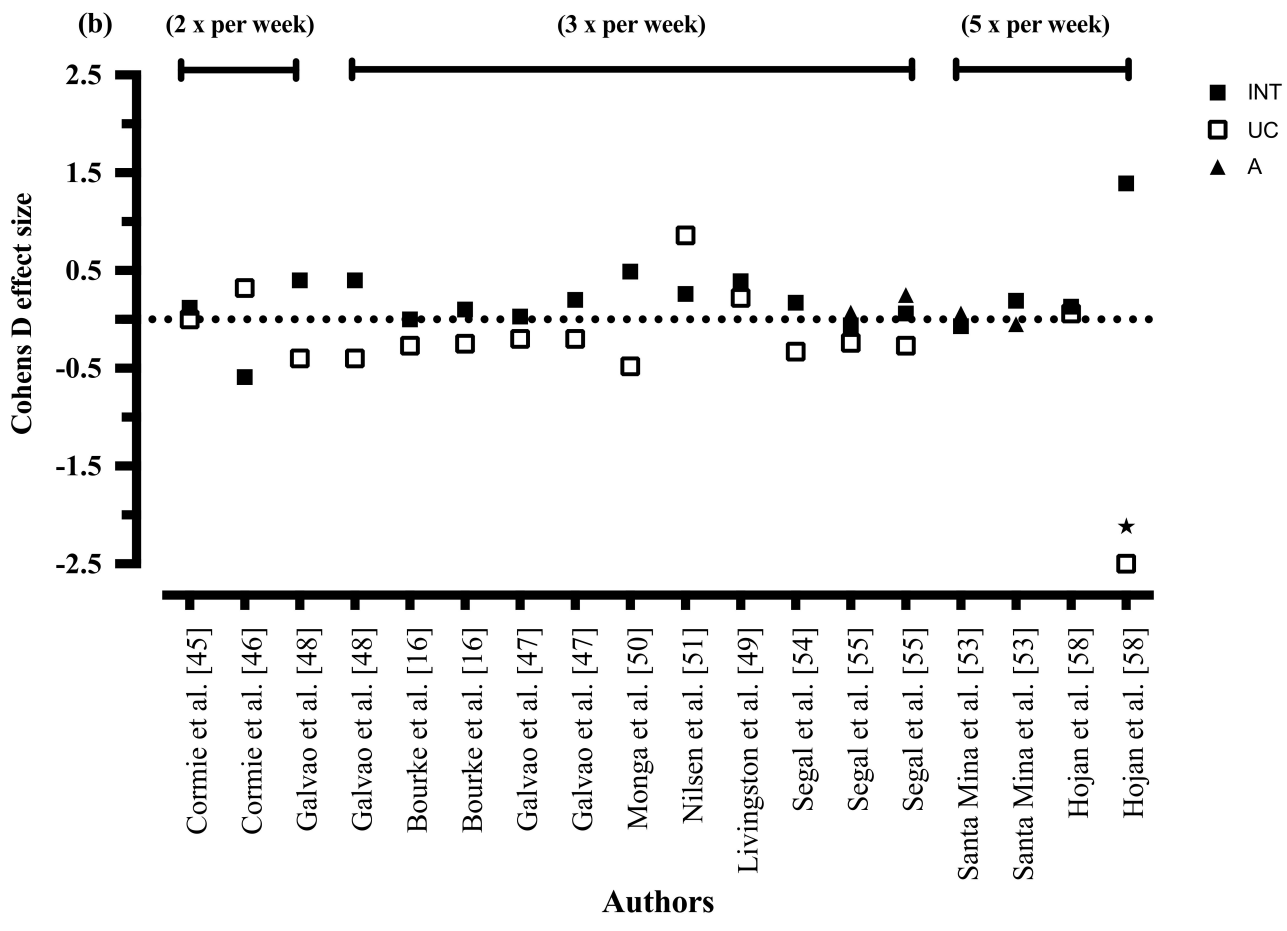

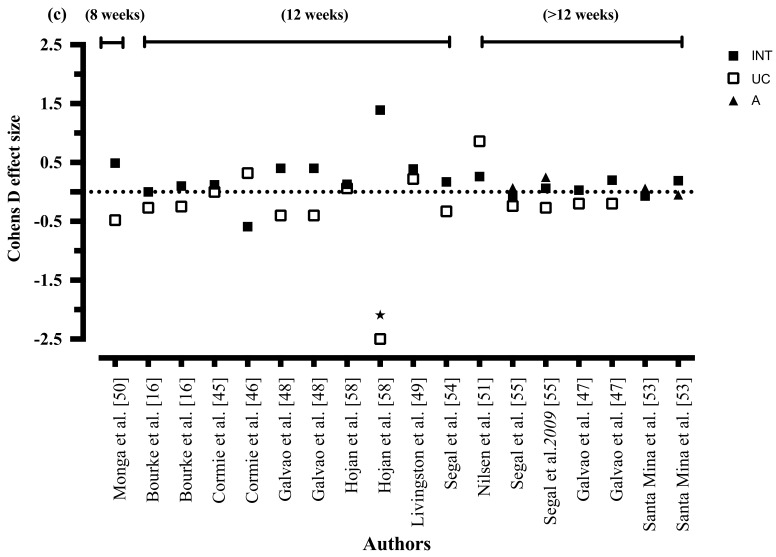
(**a**) Cohens D effect size of exercise mode on quality of life; (**b**) Cohens D effect size of exercise frequency on quality of life; (**c**) Cohens D effect size of exercise duration on quality of life (A = aerobic exercise, R = resistance training, INT = intervention, UC = usual care, b = second measure of quality of life, * = effect size of −7.04).

The effects of the interventions on quality of life are shown in Table 5. Soy supplementation demonstrated a between-group improvement in quality of life compared to a control group taking placebo supplements [56]. One of the two combined diet and exercise interventions showed between-group improvements in quality of life when compared to usual care [17]. Seven of the eleven exercise only RCTs exploring quality of life found between-groups improvement in favour of the intervention [47,48,50,53,54,55,58]. Four of eleven exercise only RCTs showed significant within-group improvements in quality of life from the exercise intervention [50,53,55,58]. The effect size of exercise mode, frequency, and duration from the intervention, compared to usual care, on quality of life are shown in Figure 3.

#### 3.8.7. Exercise Mode

Two of four combined resistance training and aerobic exercise RCTs showed between-group improvements in quality of life when compared to usual care [48], or a control group mailed the physical activity guidelines [47]. Three of five resistance training alone RCTs improved quality of life compared to usual care [54,55], or when resistance training was compared to aerobic exercise [53]. One of three aerobic exercise only RCTs [50] found between-group improvements in quality of life when compared to usual care.

#### 3.8.8. Exercise Intensity

Six of eleven moderate-vigorous (aerobic exercise) and moderate-hard (resistance training) intensity interventions showed between-group improvements compared to usual care [48,50,54,58], a control group mailed the physical activity guidelines [47], or when resistance training was compared to aerobic exercise [54]. Zero interventions exploring the effects of exercise on quality of life utilised light, high or sprint aerobic exercise, or light, very hard or maximal resistance training.

#### 3.8.9. Exercise Duration

The only intervention of 8 weeks duration showed between-group improvements in quality of life compared to usual care [50]. Three of six 12-week interventions showed between-group improvements in quality of life compared to usual care [48,54,58], whilst one intervention of 16 weeks showed no between- or within-group improvements [51]. Both interventions of 24 weeks showed between-group improvements in quality of life, with resistance training demonstrating greater improvements than aerobic exercise [37,55]. The only intervention of 52 weeks showed between-group improvements compared to a control group mailed the physical activity guidelines [47]. 

#### 3.8.10. Exercise Frequency

One of two interventions prescribing exercise twice per week showed between-group improvements in quality of life compared to usual care [48]. Four of the seven RCTs prescribing exercise three times per week showed between-group improvements in quality of life compared to usual care [50,54,55], or the physical activity guidelines [47]. Both interventions involving exercise prescribed five times per week showed between-group improvements compared to usual care [58], or when resistance training was compared to aerobic exercise [47].

## 4. Discussion

This systematic review is the first of its kind to investigate the effects of diet, exercise, and their combination on CRF and quality of life on men with prostate cancer. Soy supplements (20 g; 160 mg isoflavones) improved quality of life, but demonstrated a high number of adverse events [56]. The combination of healthy eating with aerobic exercise and resistance training reduces CRF in men treated with ADT, yet further research is needed to confirm its effects on quality of life [16,17]. Structured exercise with or without nutrition therapy appears to be beneficial to reduce CRF and improve quality of life for men with prostate cancer [16,17,45,47,48,50,53,54,55,57,58]. The individualised effects of the key exercise principles of frequency, intensity, and duration require further investigation to develop the optimal exercise prescription for effects on CRF and quality of life. The results indicate a clear literature gap in the efficacy of diet in isolation on CRF (zero studies) and quality of life (one study) in men with prostate cancer. 

### 4.1. Nutrition Therapy

The efficacy of dietary advice alone on CRF and quality of life in men with prostate cancer cannot be determined from this systematic review. Whilst soy supplementation improved prostate cancer-specific quality of life, the high dropout rate and adverse events impeded the translation of these findings to practice [56]. Furthermore, no studies have investigated the effects of nutrition therapy on CRF outcomes. Herein, a clear gap exists in the literature for the management of CRF and quality of life through nutrition therapy. Cross-sectional studies in breast cancer survivors consuming a higher quality of diet, compared to survivors with poorer diet quality, were associated with a lower prevalence of CRF [27]. Several observational studies support these cross-sectional findings, with diets high in dietary fibre (>25 g/day) [28], high in fruits and vegetables [64] and low in saturated fats [28] all independently associated with lower levels of CRF in breast cancer survivors. Importantly, a dietary intake high in fatty fish, nuts and seeds, whole grains, and vegetables was found to reduce the severity of fatigue in cancer survivors [64]; suggesting nutrition therapy warrants further research to determine its effects on CRF. In addition, nutrition management of CRF appears to be of high importance to cancer patients, with a cohort of 1290 cancer patients identifying information on the nutritional management of CRF as the second most desirable information sought after diagnosis and/or treatment (most desirable information is for a healthy diet to follow) [65]. Randomised controlled trials are required to understand the impact of diet quality, composition, dietary patterns, and subsequent utility of diet in managing CRF. 

### 4.2. Combined Nutrition Therapy and Exercise

Evidence from this systematic review indicates that fortnightly, group-based healthy eating advice with a combination of supervised aerobic and resistance training for 12-weeks can improve CRF [16,17] and may improve quality of life [16]. Improvements in CRF were observed in the two studies with fortnightly diet advice and structured supervised exercise (2–3 times per week) [16,17], whilst no significant improvement was observed in the study with a home-based intervention [52]. This difference occurred despite Bourke et al. [16,17] and O’Neil et al. [52] eliciting similar total calorie deficits (116–258 Kcal), suggesting the differences in CRF outcomes may come from the different dietary modification techniques utilised by each study. The fortnightly, group-based, educational dietary modification methods utilised in the study by Bourke et al. [16,17] were successful in alleviating CRF, whilst the one-off dietary consult utilised in the study by O’Neil and et al. [52] failed to alleviate CRF. It appears that regular interaction with a nutritionist/dietician may be more important for reducing CRF than total calorie deficit. The two diet and exercise studies demonstrating between-group improvements in CRF involved previously sedentary participants [16,17]; this, at least in part, may explain the significant improvements in CRF and quality of life following the intervention. However, it is unknown if combined diet and exercise interventions are just as effective in physically active men with prostate cancer. Irrespective of the study design and inclusion criteria, general healthy eating advice and exercise demonstrated sustained improvements in CRF at six months follow-up [16,17]; indicating diet and exercise may have the capacity to produce sustainable improvements in CRF. 

### 4.3. Exercise

This is the first review to qualitatively analyse all four of the exercise prescription principles (frequency, intensity, type, and time) on CRF and quality of life. This study is also the first to investigate the isolated effects of exercise separate to confounding adjunctive lifestyle interventions (i.e., meditation, relaxation, psychosocial support). In support of previous systematic reviews exploring the role of exercise in CRF [66] and quality of life [33,38], structured exercise has the potential to improve both CRF and quality of life. However, these findings were inconsistent, with only five of twelve interventions showing between-group improvements in CRF [45,48,55,57,58], and seven of eleven interventions showing between-group improvements in quality of life [47,48,50,53,54,55,58] in men with prostate cancer. Keogh et al. [66] suggested group-based exercise to be more beneficial than home-based exercise in reducing CRF and quality of life, yet it is unknown if other exercise prescription parameters (frequency, intensity, type and time) influence the efficacy of exercise on improving CRF and quality of life. 

Combining resistance training with aerobic exercise appears to be the most favourable mode of exercise to reduce CRF in men with prostate cancer [45,48,58]. This may, in part, explain why no studies investigating aerobic exercise saw between-group improvements in CRF [53,62], and why inconclusive findings were seen from resistance training only studies on CRF (with only two of six reporting between-group improvements) [55,57]. In contrast to the review by Keogh et al. [66], this review suggests that exercise mode may be an important prescription consideration for alleviating CRF, with resistance training with or without aerobic exercise more likely to result in improvements in CRF. This may be due to differences between reviews in inclusion criteria, with Keogh et al. [66] including studies utilising various lifestyle methods (i.e., meditation, relaxation, and psychosocial support) that may have influenced the results. In contrast to CRF, the influence of exercise mode on quality of life is less clear, with each mode (resistance training and/or aerobic exercise) reporting inconsistent benefits. Resistance training with or without aerobic exercise maybe more likely to improve quality of life compare to aerobic exercise [47,48,53,54,55,58], however this requires further exploration. 

Moderate-to-vigorous (aerobic exercise) and moderate-to-hard (resistance training) intensity exercise showed a modest effect for reducing CRF in men with prostate cancer (five of nine studies showed between-group improvements compare to usual care) [45,48,55,57,58]. To improve quality of life in men with prostate cancer, moderate intensity exercise [47,48,50,53,54,55,58] in accordance with the exercise oncology guidelines [30], appears to be of sufficient intensity. The influence of different exercise intensities on CRF and quality of life require further exploration, as none of the studies utilised light, high or sprint intensity aerobic exercise, or light, very hard or maximal intensity resistance exercise. Adherence to the prescribed intensity of exercise was poorly reported among exercise interventions. Only two of fourteen studies reported exercise intensity adherence via average heart rate or rating of perceived exertion, thus precluding our understanding of the effect of exercise intensity on CRF outcomes [46,50]. 

Six of twelve exercise programs 12 weeks or longer reported between-group improvements in CRF [45,48,55,57,58], whilst exercise interventions shorter than 12 weeks showed no between-group improvements in CRF. Exercise programs between 8 [50] and 52 weeks [37,47,48,54,55,58] showed inconsistent between-group improvements in quality of life compared to usual care. There was no apparent difference in exercise frequency on outcomes of CRF or quality of life, with between-group differences seen with 2 [48], 3 [47,50,54,55] and 5 [47,58] exercise sessions per week. No studies compared differences in exercise duration or frequency on outcomes of CRF or quality of life. 

All studies demonstrating improvements in CRF [45,48,55,57,58] and quality of life [47,48,50,53,54,55,58] were structured and supervised by a health professional. This indicates exercise physiologists, physiotherapists or other qualified exercise health professionals are an important addition to the allied health team in managing outcomes of CRF or quality of life in men with prostate cancer. 

### 4.4. Future Directions for Nutrition Therapy

There appear to be multiple pathological mechanisms that nutrition therapy can target to alter CRF and quality of life in men with prostate cancer. Exploratory literature indicates altered pro-inflammatory makers IL-6, IL-8, TNF-α, treatment-induced anaemia, hypoalbuminemia, and a reduction in lean muscle mass are associated with the prevalence of CRF [8,23,24,66,67]. Men with prostate cancer experience a high incidence of lean muscle mass loss, particularly those treated with ADT [66,68,69,70]. Preserving lean muscle mass through dietary protein intake offers an informative novel approach to alleviating CRF through dietary modification. The Nutrition and Physical Activity Guidelines for Cancer Survivors suggest dietary protein intake of 0.8 g/kg body weight to alleviate muscle decomposition [21]. Cross-sectional investigations suggest total protein intake below 1 g/kg body weight is associated with higher levels of CRF in advanced cancer patients treated with chemotherapy [29]. Nutrition therapy with a protein intake of 0.8–1.0 g/kg body weight (mean increase of 26 g/day (range: 20–34 g/day) from baseline to end of intervention) has shown significant reductions in CRF after three months in colorectal patients undergoing radiotherapy [71,72]; however, similar investigations have yet to be performed in men with prostate cancer. Further nutrition investigations are imperative to explore the relationship between protein intake, muscle mass loss, and CRF and quality of life in men with prostate cancer. 

Pro-inflammatory markers such as IL-6, IL-8, CRP and TNF-α have shown to be elevated beyond normal levels in both breast and prostate cancer patients experiencing CRF [23,24]. Cross-sectional evidence suggests a higher intake of anti-inflammatory and antioxidant nutrients decreases CRF in participants with breast cancer when compared to lower intakes [64]. Dietary modification involving high intake of fruits, vegetables, whole grains, and oily fish (high antioxidant and anti-inflammatory properties) has been shown to significantly reduce CRF within three months compared to general healthy eating in breast cancer survivors [73]. Dietary intake of anti-inflammatory properties associated with fish (omega-3) has previous been associated with reductions in CRF [74,75]; further supporting the theorised effects of a high anti-inflammatory diet on CRF. Recently, much attention has turned to a Mediterranean diet [a high intake of plant foods (fruits and vegetables), cereals and whole grain breads, pulses, nuts and seeds, olive oil as a main source of fat, a moderate amount of cheese and yoghurt, low qualities of red meat and a moderate amount of fish] due to the high anti-inflammatory capacity of the diet [76]. A Mediterranean diet pattern in high risk cardiovascular populations has been shown to significantly reduce pro-inflammatory markers associated with CRF, such as CRP, IL-6 and IL-8 after 3–12 months [77,78]. Furthermore, men with prostate cancer have shown high adherence following adoption of a Mediterranean diet [63]; yet the effects of the Mediterranean diet on outcomes such as CRF and quality of life in men with prostate cancer are unknown. Structured and individualised dietary modification aligned with a Mediterranean diet appears to offer a plausible evidence-based alleviation to the theorised mechanisms underpinning CRF. Dietary intake high in anti-inflammatory capacity, such as a Mediterranean diet, warrants future investigation to provide an evidence-based nutrition practice for men with prostate cancer experiencing CRF. 

Weight status, particularly obesity (Body Mass Index (BMI) > 30 kg/m^2^), is thought to be a modifiable risk factor for the prevalence of CRF [24]. Up to 70% of men diagnosed with prostate cancer and/or post-treatment for prostate cancer are of an overweight or obese BMI classification [25,79]; this may explain, at least in part, the high prevalence of CRF during and/or after prostate cancer treatment. Substantial longitudinal evidence exists in breast cancer survivors suggesting an overweight/obese BMI has a temporal association with CRF [80]. Cross-sectional evidence in breast cancer indicate women with a BMI > 30 kg/m^2^ reported higher levels of CRF than women with a BMI < 30 kg/m^2^ [81]; however, evidence specifically in men with prostate cancer is scarce. A greater percentage of body fat (mean body fat percentage of 34%) was a significant indicator of CRF in women with a cohort of breast cancer survivors [82], supporting the inclusion of body composition analysis in men with prostate cancer when exploring CRF outcomes. Dietary weight loss and outcomes of CRF in prostate cancer offers promise, with a recent systematic review in prostate cancer indicating dietary weight loss interventions elicit reductions of −0.8 to −6.1 kg (median −4.5 kg) [41]. Weight loss, and in particular reductions in fat mass, may also reduce pro-inflammatory markers associated with CRF. High abdominal visceral fat, and an obese BMI classification are known to create pro-inflammatory state through altered cytokines IL-6, IL-8 [83], and TNF-α [84]. Dietary induced weight loss in non-oncological populations has shown reductions in inflammatory cytokines (IL-6 and IL-8) associated with CRF [85,86,87,88], suggesting weight loss in obese men with prostate cancer to be a plausible and measurable nutrition therapy outcome. Considering the theorised mechanisms contributing to CRF, understanding the role of targeted nutrition therapy in altering these theorised CRF mechanisms may provide novel insight into the optimal management of CRF and quality of life of men with prostate cancer. 

### 4.5. Recommendations

This systematic review revealed a lack of studies in the area of dietary intervention for men treated with prostate cancer. To advance the field of dietetic-oncology, future investigations in this area should focus on overall dietary patterns rather than individual nutrients to manage the vast array of side effects from prostate cancer treatment, including CRF and quality of life. Incorporating measures of lean muscle mass, fat mass and inflammatory cytokines when exploring the dietetic management of CRF will enhance our understanding of the potential mechanisms through which diet may alleviate CRF. A targeted diet and exercise approach may elicit a multi-faceted effective strategy to improve CRF and quality of life through influencing the different pathological pathways contributing to CRF. 

There are several limitations of this systematic review worthy of comment. CRF and quality of life questionnaires are predominantly tertiary outcome measures in this literature. This limits our ability to ascertain the optimal nutrition therapy and exercise prescription for improving CRF and quality of life. Furthermore, the heterogeneity in quality of life assessment tools precludes meaningful comparisons and conclusions at present. Future exercise-oncology research should examine difference in intensity, frequency of exercise, type and duration of exercise, to determine the optimal exercise prescriptive approach to managing CRF and quality of life. Consistently reporting exercise intensity via a heart rate monitor and/or rating of perceived exertion will help determine the influence of exercise intensity and contribute to our understanding of the optimal ‘dose’ of exercise for improving CRF and quality of life in men with prostate cancer. Finally, whilst this study attempted to explore the effects of exercise on CRF and quality of life on men who had undergone any prostate cancer treatment, none of the studies comprised within this review included men undertaking chemotherapy. Therefore, the effects of exercise on CRF and quality of life in men with prostate cancer treated with chemotherapy are yet to be determined. 

## 5. Conclusions

There is a clear literature gap in the efficacy of diet interventions on prostate CRF and quality of life. Although soy supplementation has shown improvements in prostate cancer-specific quality of life, the translation of these results to practice is limited due to the high adverse events and dropout rates. Consuming a diet aligned with the healthy eating guidelines, and exercising at a moderate-vigorous (aerobic exercise) and/or moderate-to-hard (resistance training) intensity may reduce CRF; yet outcomes on quality of life warrant further investigation. Structured and supervised exercise positively influences both CRF and quality of life in men with prostate cancer. However, there was considerable methodological heterogeneity in all aspects of study design and endpoints, making further exercise prescription recommendations difficult. 

Future dietetic- and/or exercise-oncology studies should aim to investigate the mechanistic underpinnings of the intervention on CRF and quality of life. Individualised nutrition therapy to target protein intake for maintenance of lean muscle mass, energy density for reducing total body mass, and/or anti-inflammatory diets for reducing systemic inflammation, may offer controlled and targeted approaches to future dietetic research aiming to improve CRF in men with prostate cancer. Whilst exercise appears to improve CRF and quality of life, identifying the impact of the key exercise prescription principles, through structured RCTs, will help establish evidence-based guidelines for the role of exercise prescription in managing CRF and quality of life in men with prostate cancer. 

## Figures and Tables

**Table 1:** Description of the Population, Intervention, Control, Outcome (PICO) criteria.

Criteria	Description
Participants	Men with a histologically confirmed diagnosis of prostate cancer (including all stages of, and treatments for, prostate cancer)
Intervention(s)	Any structured diet only intervention
	Any exercise only protocol detailing frequency, intensity, time and type of exercise
	Any combined diet and exercise protocol, for any duration (with the exception of a single bout of exercise comparing pre- and post-exercise acute fatigue measures)
Comparison(s)	Comparison group receiving diet, exercise, or a combined diet and exercise of a lesser intensity, or a control group not receiving the intervention at any time point during the trial
Outcome(s)	Changes in cancer-related fatigue and quality of life

**Table 2:** Delphi quality rating table.

	Author	1. Randomisation	2. Treatment Allocation	3. Group Similarity at Baseline, or Adjustment in Analysis	4. Eligibility Criteria Specified	5. Point Estimates and Measures of Variability	6. Intention to Treat Analysis	Total Score
1	Bourke, et al. [16]	Y	Y	Y	Y	Y	Y	100%
2	Bourke, et al. [17]	Y	Y	Y	Y	N	N	66%
3	Cormie, et al. [46]	Y	Y	Y^a^	Y	Y	Y	100%
4	Cormie, et al. [45]	Y	Y	Y	Y	Y	Y	100%
5a	Galvão, et al. [48]	Y	Y	Y	Y	Y	Y	100%
5b	Buffart, et al. [61] ^b^							
6a	Galvão, et al. [47]	Y	Y	Y	Y	Y	Y	100%
6b	Buffart, et al. [60] ^b^							
7	Hojan, et al. [58]	Y	Y	Y	Y	Y	N	83%
8	Livingston, et al. [49]	Y	N	Y	Y	Y	N	66%
9	McQuade, et al. [59]	Y	Y	U	Y	N	N	50%
10	Monga, et al. [50]	Y	U	Y	Y	Y	N	66%
11	Nilsen, et al. [51]	Y	Y	Y	Y	Y	Y	100%
12	O’Neil, et al. [52]	Y	Y	Y	Y	Y	N	83%
13	Santa Mina, et al. [53]	Y	Y	Y	Y	Y	Y	100%
14	Segal, et al. [54]	Y	Y	Y	Y	Y	Y	100%
15	Segal, et al. [55]	Y	Y	Y ^a^	Y	Y	Y	100%
16	Truong, et al. [62]	N	N	Y	Y	N	U	33%
17	Vitolins, et al. [56]	Y	Y ^a^	U	Y	N	Y	66%
18	Winters-Stone, et al. [57]	Y	Y	Y	Y	N	Y	83%
Number of papers scoring a point/total papers		17/18	15/18	16/18	18/18	14/18	11/18	

Y, yes; N, no; U, unclear after requesting information from the authors; ^a^ Quality rated after requesting and obtaining additional information from the study authors; ^b^ Same trial, but different analysis-of-questionnaire outcomes are reported.

**Table 3:** Study characteristics.

Author (Year) Country	Study Design	Participants (Mean Age ± SD Range (Years)	Prostate Cancer Treatment (Treatment Duration ± SD Range (Months))	Control Group	Dropout Number	Exercise				Nutrition Therapy	
						Mode	Time/Intensity	Frequency	Duration	Intervention Details (Frequency, Delivery, Diet)	
Combined Diet and Exercise Interventions											
Bourke, et al. [16] United Kingdom	RCT	I = 25 (71.3; 6.4)	ADT (30 ± 31)	Y = usual care	I = 4	A and R	A: 55–80% APMHR	3 times per week	12 weeks	Fortnightly small group nutrition seminars, with nutrition handout pack. Diet composition of low SF, refined CHO, moderate EtOH, high fibre, fruit and vegetables.	
		C = 25 (72.2; 7.7)			C = 3		R: progressive resistance load of 2–4 sets of 8–12 repetitions of upper and lower body muscle groups (unknown amount of exercises)				
Bourke, et al. [17] United Kingdom	RCT	I = 50 (71; 6)	ADT (33 ± 33)	Y = usual care	I = 7	A and R	A: 55–80% APMHR	3 times per week	12 weeks	Fortnightly small group nutrition seminars, with nutrition handout pack. Diet composition of low SF, refined CHO, moderate EtOH, high fibre, fruit and vegetables.	
		C = 50 (71; 8)			C = 8		R: progressive resistance load of 2–4 sets of 8–12 repetitions of upper and lower body muscle groups (unknown amount of exercises)				
O’Neil, et al. [52] United Kingdom	RCT	I = 47 (69.7; 6.8)	ADT (26.4 ± 32.4)	Y = usual care	I = 1	A	Moderate: intensity NM	3 times per week	24 weeks	Baseline consult to individually meet ≥5 servings of vegetables and fruits/day, 30–35% of total energy from fat/day, ≤10% energy from SF/day, 10% energy from PUFA/day, limited consumption of processed meats, 25–35 g fibre/day, limited EtOH, and intake of Na^+^, and/or sugar.	
		C = 47 (69.9; 7)			C = 2						
Diet Only Interventions											
Vitolins, et al. [56] United States	RCT	N = 78	Orchiectomy, LHRH, Antiandrogen, Radiation	N	Group 1 = 39				12 weeks	Participants randomly assigned to received one of four treatments: (Group 1) placebo pill or 75 mg venlafixine once daily with 20 g soy protein containing 160 mg isoflavones, (Group 2) 75 mg venlafaxine or placebo once daily without soy protein155	
		(Group 1) = 30 (67; Range 47–81)									
		(Group 2) = 30 (67; Range 47–82)			Group 2 = 39						
		(Group 3) = 30 (71; Range 54–85)									

		(Group 4) = 30 (69; Range 46–91)									
Exercise Only Interventions											
Cormie, et al. [45] Australia	RCT	I = 32 (69.9; 6.5)	ADT I = 6.2; 1.6 (days)	Y = usual care	I = 1	R and A	A: 20–30 min 70–85% estimated HR_max_	2 times per week	12 weeks		
		C = 31 (67.1; 7.5)	C = 5.6: 2.0 (days)		C = 7		R: Progressive resistance load, 8 exercises 2–4 sets of 6–12 repetitions, 60–85% 1RM				
Cormie, et al. [46] Australia	RCT	I = 10 (73.1; 7.5)	ADT, radiation (NA)	Y = usual care	I = 2	R	Progressive resistance load, 8 exercises 2–4 sets of 8–12 repetitions, 60–85% 1RM	2 times per week	12 weeks		
		C = 10 (71.2; 6.9)			C = 3						
Galvão, et al. [47]; Buffart, et al. [60] Australia	RCT	I = 50 (71.9; 5.6)	ADT and radiation	Y = mailed exercise guidelines	I = 14	R and A	A: 20–30 min at 70–85% HR_max_	3 times per week	52 weeks		
			I = 12.9; 5.9				R: Progressive resistance load, 8 exercises 2–4 sets of 12 repetitions (moderate intensity)				
		C = 50 (71.5; 7.2)	C = 11.0; 5.9		C = 8		C: modified educational booklet to perform 150 min per week of moderate PA				
Galvão, et al. [48]; Buffart, et al. [61] Australia	RCT	I = 29 (69.5; 7.3)	ADT I = 18.2; 38.5	Y = usual care	I = 1	R and A	A: 15–20 min 70–80% HR_max_	2 times per week	12 weeks		
		C = 28 (70.1; 7.3)	C = 10.1; 26.8		C = 1		R: Progressive resistance load, 8 exercises 2–4 sets of 6–12 repetitions (moderate intensity)				
Hojan, et al. [58] Poland	RCT	I = 27 (67.4; 8.3)	ADT, and Radiation (NA)	Y = usual care	NA	R and A	A: 30 min 65–70% estimated maximal heart rate (220-age (years))	5 times per week	12 weeks		
		C = 27 (69.9; 7.2)					R: 2 sets of 8 repetitions at 70–75% estimated 1RM of upper and lower body muscle groups				
Livingston, et al. [49] Australia	RCT	I = 54 (66.9; 8.2)	Radical prostatectomy, radiation, ADT (NA)	Y = usual care	I = 7	R and A	A: 20 mins 40–70% APMHR	2 supervised, 1 home-based per week	12 weeks		
		C = 93 (64.7; 8.7)			C = 10		R: progressive resistance load, 4–8 exercises 2 sets of 8–12 repetitions (moderate intensity)				
							Unsupervised: body weight and Thera-band exercises				
McQuade, et al. [59] United States	RCT	I = 26 (65; 5.9)	Radiation (NA)	Y = usual care	I = 5	R	40 min of 8–12 sets of 8–12 repetitions of various muscle groups (light intensity)	3 times per week	8 weeks		
		U = 24 (66; 8.4)			U = 0						
		Tai chi = 26 (62.2; 7.4)			Tai chi = 5						
Monga, et al. [50] United States	RCT	I = 11 (68; 4.2)	Radiation	Y = usual care	N = 9	A	30 min at (0.65) × (HR_max_ − resting HR) + resting HR, with 15–20 min warm-up and cool-down	3 times per week	8 weeks		
		C = 10 (70.6; 5.3)									
Nilsen, et al. [51] Norway	RCT	I = 28 (66; 54–76)	ADT I = 17 ± 87 C = 18 ± 8.2	Y = usual care	I = 6	R	Progressive resistance load, 9 exercises 2–3 sets of 10 repetitions, 40–90% 1RM	3 times per week	16 weeks		
		C = 30 (66; 54–76)	Radiation I & C = 3.0 ± 1.3		C = 3						
Santa Mina, et al. [53] Canada	RCT	A: 32 (72.1; 8.9)	ADT (NA)	N	A: 13	A or R	A: 30–60 min 60–80% HRmax	3–5 times per week	24 weeks		
		R: 34 (70.6; 9.5)			R: 22						
					C: 1		R: Progressive resistance load, 11 exercises 2–3 sets, 8–12 repetitions (moderate intensity)				
Segal, et al. [54] Canada	RCT	I = 82 (68.2; 7.9)	ADT I = (12.5; 18.9)	Y = waiting list	I = 8	R	Progressive resistance load, 9 exercises, 2 sets of 8–12 repetitions, 60–70% 1RM	3 times per week	12 weeks		
		C = 73 (67.7; 7.5)	C = (13.4; 22.2)		C = 12						
Segal, et al. [55] Canada	RCT	A: 40 (66.2; 6.8)	Radiation ± ADT (NA)	Y = usual care	A: 3	A or R	A: Progressive HR workload of (weeks 1–4) 50–60% VO_2peak_ to (weeks 5–24) 70–75% VO_2peak_	3 times per week	24 weeks		
		R: 40 (66.4; 7.6)			R: 7		R: Progressive resistance load, 11 exercises 2–3 sets, 8–12 repetitions, 60–70% 1RM				
		C: 41 (66.3; 7.0)			C: 1						
Truong, et al. [62] Canada	Prospective Cohort	I = 50 (67; 6.5)	Radiation ± ADT I = 12; 2.9	Y = usual care	I = 8	A	A: 20 min at 60–70% APMHR	3 times per week	12 weeks		
		C = 30 (69; 6.3)	C = 12; 2.8		C = 0						
Winters-Stone, et al. [57] United States	RCT	R = 29 (69.9; 9.3)	ADT ± Radiation I = (39.0; 36.1)	N	I = 3	R	R: progressive resistance load per % BW, 8 exercises 1–2 sets 8–14 repetitions (moderate intensity)	3 times per week	52 weeks		
		Stretching = 22 (70.5; 7.8)	C = (28.5; 29.2)		C = 5						

ADT = androgen deprivation therapy, A = aerobic exercise, APMHR = age predicted maximum heart rate, BW = body weight, C = control group, CHO = carbohydrate, EtOH = alcohol HR = heart rate, HRmax = maximum heart rate, I = intervention, LHRH = Luteinizing Hormone-Releasing Hormone, MPP = milk protein powder, NA^+^= sodium, NM = not mentioned, N = no, PA = physical activity PUFA = polyunsaturated fatty acid, RCT = randomised controlled trial, RM = repetition maximum, R = resistance training, SF = saturated fat, SP = soy protein, SD = standard deviation, V = venlafaxine, Y = yes.

**Table 4:** Cancer-related fatigue outcome scores.

Author (Year) Country	Measure of Fatigue	Baseline	Outcome Measure ± SD		Δ Fatigue Pre- and Post- Intervention (Mean ± SD (95% CI))		Δ Fatigue Pre-Intervention Follow-up (Mean ± SD (95% CI))	
			Post Intervention	Follow-up	Between-Group	Within-Group	Between-Group	Within-Group
Bourke, et al. [16] United Kingdom	FACT-F	I = 44 ± 6	48 ± 4	❖ 43 ± 7	5.4 * (0.8, 10.0)		❖ 3.1 * (−0.3, 6.4)	
		C = 42 ± 8	48 ± 4	40 ± 8	(*p* = 0.002)		(*p* = 0.006)	
Bourke, et al. [17] United	FACT-F	I = 40.3 ± 8.2	45.8 (NA)	43.5 (NA)	5.3 * (2.7, 7.9)		❖ 3.9 * (1.1, 6.8)	
		C = 41.4 ± 8.6	42.4 (NA)	41.9 (NA)	(*p* ≤ 0.001)		(*p* = 0.007)	
O’Neil, et al. [52] United Kingdom	MFSI-SF	I = 30.7 ± 14.9	29.4 ± 15.5		2.8 (−7.8, 2.1)			
		C = 32.8 ± 17.6	34.1 ± 19		(*p* = 0.26)			
Vitolins, et al. [56] United States								
Cormie, et al. [45] Australia	FACT-F	I = 43.7 ± 8.3	I = 43.8 ± 6.8		3.1 * (0.1, 6.2)	I = 0.1 ± 6.6 (*p* = 0.961) C = −3.4 (6.4) * (*p* = 0.006)		
		C = 44.8 ± 8.5	C = 41.4 ± 9.5		(*p* = 0.042)			
Cormie, et al. [46] Australia	MFSI-SF	I = 5.2 ±16.8	I = 8.8 ± 24.9		−4.2 (−17.6, 9.2) (*p* = 0.521)			
		C = 6.0 ± 12.3	C = 3.8 ± 13.7					
Galvão, et al. [47]; Buffart, et al. [60] Australia								
Galvão, et al. [48]; Buffart, et al. [61] Australia	EORTC-30 (F)	I = 16.8 ± 17	I = 14.6 ± 13.8		10.08 * (−18.33, 1.82)			
		C = 29.7 ± 18.3	C = 30.6 ± 17.6					
Hojan, et al. [58] Poland	FACT-F	I = 42.7 ± 2.1	I = 43.9 ± 5.0		19.2 ± 4.7 * (*p* < 0.01)	I = 1.2 ± 4.8		
		C = 42.5 ± 2.5	C = 24.7 ± 4.5			C = −17.8 ± 3.7 * (*p* < 0.01)		
	EORTC-C30 (F)	I = 27.3 ± 19.7	I = 30.7 ± 21.4		11.2 ± 22.6 * (*p* < 0.05)	I = 3.4 ± 19.3		
		C = 28.0 ± 21.9	C = 42.1 ± 23.6			C = 14.0 ± 17.8 * (*p* < 0.05)		
Livingston, et al. [49] Australia								
McQuade, et al. [59] United States	BFI	I = 1.47 ± 0.39	I = 1.65 ± 0.38					I = 2.38 ± 0.42
		C = 1.97 ± 0.34	C = 1.87 ± 0.33					C = 1.81 ± 0.35
Monga, et al. [50] United States	PFS	I = 2.4 ± 2.4	I = 0.8 ± 1.8		−4.3 ± 2.1 (*p* = 0.001)	I = −1.6 ± 2.0 * (*p* = 0.02)		
		C = 1.1 ± 1.9	C = 3.8 ± 2.2			C = 2.7 ± 2.2 (*p* = 0.004)		
Nilsen, et al. [51] Norway	EORTC-30 (F)	I = 34.5 ± 15.2	I = 33.7 ± 16.1		2.3 (−5.84, 10.54)	I = −0.8 (−6.41, 4.82)		
		C = 36.5 ± 14.9	C = 33 ± 22.3		(*p* = 0.568)	C = −3.5 (−9.74, 2.70)		
Santa Mina, et al. [53] Canada	FACT-F	A: 42 ± 8.4	A: 41.4 ±1.4	A❖: 42.2 ± 1.3 A♦: 42.4 ± 1.4	*p* = 0.795❖	A❖: 0.19 (0.95) A♦: 0.35 (1.27)		
		R: 38.1 ± 12.1	R: 38.7 ± 1.7	R❖: 35.6 ± 2.2 R♦: 37.9 ± 2.2	*p* = 0.767♦	R❖: 2.06 (1.94) R♦: 2.83 (1.83)		
Segal, et al. [54] Canada	FACT-F	I = 40.8 ± 10.6	I = 41.6 ± 10.5		I = (*p* = 0.002)	I = 0.8 ± 5.8 C = -2.2 ± 5.8		
		C = 42.5 ± 8.5	C = 40.3 ± 9.4					
Segal, et al. [55] Canada	FACT-F	A: 44.1 ± 8.7	A: 44.2 ± 8.9		A: 2.65 (−0.29 ± 5.58) (*p* = 0.80)	A: 0.2 (−1.9 ± 2.29) (*p* = 0.850)		
		R: 42.8 ± 8.7	R: 45.1 ± 9.1		R: 4.78 * (1.77 ± 7.78) (*p* = 0.002)	R: 2.33 * (0.13 ± 4.53) (*p* = 0.040)		
		C: 44.6 ± 8.7	C: 42.1 ± 8.8			C: −2.45 * (−4.50, −0.40) (*p =* 0.020)		
Truong, et al. [62] Canada	BFI	I✜ = 6.3	I✜ = 6.5	I✜ = 6.2	*(p* = 0.40)			
		C✜ = 4.7	C✜ = 9.0	C✜ = 9.6				
Winters-Stone, et al. [57] United States	SCFS	I = 9.87 ± 4.47	I = 9.22 ± 3.46	♦ I = 8.83 ± 3.19	*p* < 0.01			
		C = 9.92 ± 3.58	C = 9.17 ± 2.98	C = 9.83 ± 3.66				

A = aerobic exercise BFI = brief fatigue inventory, C = control group, EORTC = European organisation for research and treatment of cancer, F = fatigue FACT = the functional assessment of cancer treatment, FSS = fatigue severity scale; a change score less than 0 represents reductions in fatigue, MFSISF = multidimensional fatigue symptom inventory-short form, PFS = Piper fatigue scale, R = resistance training, SCFS = Schwartz cancer fatigue scale, ❖= follow-up data at 6 months, ♦= follow-up data at 12 months, ✜ = data presented as mean scores only. * = significant change *p* < 0.05. NOTE: FACT-F, EORTC-Q30, scale; a change score greater than 0 represents a reduction in fatigue; PFS, SCFS, BFI, MFSI-SF scale: a change score greater than 0 represented higher fatigue.

**Table 5:** Quality of life outcome scores.

Author (Year) Country	Measure of Quality of Life	Baseline	Outcome Measure ± SD		Δ Quality of Life Pre- and Post- Intervention (Mean ± SD (95% CI))		Δ Quality of Life Pre-Intervention Follow-up (Mean ± SD (95% CI))	
			Post Intervention	Follow-up	Between-Group	Within-Group	Between-Group	Within-Group
Bourke, et al. [16] United Kingdom	FACT-G	I = 91 ± 10	91 ± 10	❖ 90 ± 13	3.6 (−3.9, 11.0)		❖ 1.8 (−1.5, 8.6)	
		C = 89 ± 13	86 ± 18	87 ± 17	(*p* = 0.25)		(*p* = 0.36)	
	FACT-P	I = 127 ± 13	128 ± 14	❖ 125 ± 20	5.5 (−4.2, 15.3) (*p* = 0.21)		❖ 1.0 (−8.6, 10.6)	
		C = 125 ± 19	121 ± 25	NA			(*p* = 0.45)	
Bourke, et al. [17] United Kingdom	FACT-P	I = 121.8 ± 15.6	(NA)	(NA)	8.9 * (3.7, 14.2)		❖ 3.3 (−2.6, 9.3)	
		C = 119.9 ± 21.3			(*p* = 0.001)		(*p* = 0.55)	
	FACT-P	I = 33.7 ± 7.4	I = 118 ± 21.1		2.8 (−1.3, 6.9)			
		C = 34.2 ± 7.54	C = 117.5 ± 22.6		(*p* = 0.2)			
O’Neil, et al. [52] United Kingdom								
Vitolins, et al. [56] United States	FACT-P				I = 112.5 ± 6.0 (*p* = 0.048) *			
					C = 103.8 ± 6.2			
	FACT-G				I = 81.9 ± 4.3 (*p* = 0.025)			
					C = 74.1 ± 4.5			
Cormie, et al. [45] Australia	SF-36	I = GH: 53.6 ± 9.1	I = GH: 54.4 ± 10.4		1.0 (−2.3, 4.3) (*p* = 0.554)	I = 0.8 ± 6.4 (*p* = 0.468)		
		C = GH: 52.8 ± 7.8	C = GH: 52.8 ± 8.5			C = 0.0 ± 6.7 (*p* = 0.998)		
Cormie, et al. [46] Australia	SF-36	I = GH: 45.6 ± 10	I = GH: 41.7 ± 8.6		1.9 (−4.1, 7.9)			
		C = GH: 42.4 ± 8.6	C = GH: 44.5 ± 9.8		(*p* = 0.508)			
Galvão, et al. [47]; Buffart, et al. [60] Australia	SF-36	I = GH: 51.5 ± 9.9	I = GH: 51.3 ± 9.5	I = GH: 49.2 ± 12.5	1.3 (−0.5, 3.1)		−1.7 (−4.7, 1.2)	
		C = GH: 49.8 ± 8.3	C = GH: 48.5 ± 10.0	C = GH: 49.3 ± 9.9	(*p* = 0.167)		(*p* = 0.242)	
	EORTC-C30	I = GH: 77.3 ± 16.7	I = GH: 79.1 ± 13.6	I = GH: 76.9 ± 16	7.39 * (2.06, 12.73)		2.87 (−1.00–6.73)	
		C = GH: 78.5 ± 16.0	C = GH: 75.8 ± 22.4	C = GH: 75.0 ± 17.8				
Galvão, et al. [48]; Buffart, et al. [61] Australia	SF-36	I = GH: 66.0 ± 23.1	I = GH: 71.4 ± 17.5		12.9 * (1.9, 23.9)			
		C = GH: 67.3 ± 23.1	C = GH: 60.2 ± 26.7		(*p* = 0.022)			
Hojan, et al. [58] Poland	EORTC-30	I = GH: 53.7 ± 18.2	I = GH: 55.4 ± 19.9		0.32 ± 18.9	I = GH: 1.7 ± 27.7		
		C = GH: 54.2 ± 23	C = GH: 55.1 ± 17.7			C = GH: 0.9 ± 21.1		
	FACT-G	I = 70.7 ± 2.1	I = 72.3 ± 6.3		17.8 ± 5.9 * (*p* < 0.05)	I = 1.6 ± 4.8		
		C = 70.0 ± 1.9	C = 54.4 ± 3.9			C = −15.6 ± 2.9 * (*p* < 0.01)		
Livingston, et al. [49] Australia	EORTC-30	I = GH: 75.9 ± 17.4	I = GH: 80.3 ± 14.7		2.2 (−2.6, 6.9)			
		C = GH: 77.5 ± 16.0	C = GH: 80.0 ± 15.9		(*p* = 0.37)			
McQuade, et al. [59] United States								
Monga, et al. [50] United States	FACT-P	I = 138.5 ± 24.1	I = 145.9 ± 18.3		13.8 * ± 10.1	I = 7.4 * ± 10.4 (*p* = 0.04)		
		C = 144.5 ± 9.2	C = 138.1 ± 12.7		(*p* = 0.006)	C = −6.4 ± 9.8 (*p* = 0.07)		
Nilsen, et al. [51] Norway	EORTC-30	I = GH: 76.5 ± 17.3	I = GH: 79.6 ± 17		−6.9 (−13.9, 0.1)	I = 3.1 (−1.12, 7.29) C = 12.2 (6.54, 17.93)		
		C = GH: 66.7 ± 19.6	C = GH: 78.9 ± 20.7		(*p* = 0.054)			
Santa Mina, et al. [53] Canada	FACT-P	A: 123.9 ± 17.3	A: 124.4 ± 3.1	A❖: 124.2 ± 3.1 A♦: 125.5 ± 3.0	*p* = 0.796❖	A❖: 0.52 (2.48) A♦: 1.61 (2.86)		
		R: 119.3 ± 19.6	R: 118.6 ± 3.4	R❖: 117.4 ± 4.1 R♦: 119 ± 4.4	*p* = 0.207♦	R❖: 2.68 (3.83) R♦: 6.11 (4.72)		
	PORPUS	A: 67.3 ± 11.5	A: 67.0 ± 1.2	A❖: 65.8 ± 2.1 A♦: 67.2 ± 2.0	p = 0.434❖	A❖: −2.35 (1.82) A♦: −0.33 (1.93)		
		R: 62.2 ± 10.4	R: 63.2 ± 1.9	R❖: 62.3 ± 2.2 R♦: 64.5 ± 2.8	*p* = 0.021♦	R❖: −0.22 (1.83) R♦: 8.09 * (2.45)		
Segal, et al. [54] Canada	FACT-P	I = 118.2 ± 16.7	I = 120.2 ± 15.9		I = (*p* = 0.001)	I = 2.0 ± 9.1 * C = −3.3 ± 10.2		
		C = 120.9 ± 13.6	C = 117.6 ± 14.9					
Segal, et al. [55] Canada	FACT-P	A: 37.5 ± 6.4	A: 37.8 ± 6.5		A: 1.44 (−0.8, 3.68) (*p* = 0.088)	A: 0.31 (−1.29, 1.90) (*p* = 0.703)		
		R: 37.4 ± 6.4	R: 37.7 ± 6.7		R: 1.40	R: 0.27		
		C: 37.1 ± 6.4	C: 36.0 ± 6.4		(−0.89, 3.7) (*p* = 0.22)	(−1.41, 1.95) (*p* = 0.750) C: −1.13 (−2.70, 0.43) (*p* = 0.154)		
	FACT-G	A: 89.5 ± 13	91.8 ± 13.1		A: 2.35 (−0.06 ± 4.77) (*p* = 0.055)	A: 2.52 (−0.85–5.9) (*p* = 0.141)		
		R: 91.8 ± 13.1	92.4 ± 13.4		R: 4.17 * (1.62 ± 6.7) *p* = (0.002)	R: 4.34* (0.88 ± 7.8) (*p* = 0.015)		
		C: 90.0 ± 130	C: 87.5 ± 13.2			C: −0.17 (−2.53, 2.19) (*p* = 0.886)		
Truong, et al. [62] Canada								
Winters-Stone, et al. [57] United States								

A = aerobic exercise, C = control, EORTC = European organisation for research and treatment of cancer, FACT = the functional assessment of cancer treatment, G = general, GH = general/global health score, I = intervention, P = prostate, PORPUS = patient-oriented prostate utility scale, R = resistance training, SF = the medical outcome study 36-item short-form 36 health survey. ❖= follow-up data at 6 months, ♦= follow-up data at 12 months, * = *p* < 0.05. NOTE: in all questionnaires, a change score greater than 0 represents an improvement in prostate-specific quality of life.
